# Possible Steps of the Carboxylation of Ribulose-1,5-biphosphate from Intermediates: 2,3-Enediol versus 1,2-Enol

**DOI:** 10.3390/ijms22189749

**Published:** 2021-09-09

**Authors:** Roman G. Fedunov, Victor A. Sokolov

**Affiliations:** 1 V.V. Voevodsky Institute of Chemical Kinetics and Combustion/Laboratory of Photochemistry, Institutskaya Str. 3, 630090 Novosibirsk, Russia; fedunov@kinetics.nsc.ru; 2 Institute of Molecular and Cellular Biology/Plant Apomixis Laboratory, Acad. Lavrentiev Ave. 8/2, 630090 Novosibirsk, Russia

**Keywords:** photosynthesis, Rubisco, enolization, carboxylation

## Abstract

Ribulose 1,5-bisphosphate (RuBP) undergoes enolization to initiate fixation of atmospheric carbon dioxide in the plant carbon cycle. The known model assumes the binding of RuBP to the Rubisco active site with the subsequent formation of 2,3-enediol (2,3,4-trihydroxypent-2-ene-1,5-diyl diphosphate). In the present study, it is assumed that 1,2-enol (2,3,4-trihydroxypent-1-ene-1,5-diyl diphosphate) can be formed in the enolization step to initiate the carboxylation reaction. We have used Kohn–Sham density functional theory on WB97X-D3/Def2-TZVP levels to compare the reaction barriers in the two ways. We considered the pathways of carboxylation of 1/2-ene (mono/di)ol via the C1 and C2 carbons without taking into account the binding of RuBP to the magnesium ion. Calculations of Gibbs free energies confirm the equal probability of the formation of 2,3-enediol and 1,2-enol. Quantum–chemical modeling of enolization and carboxylation reactions supports the important role of the bridging water molecule and diphosphate groups, which provide proton transfer and lower reaction barriers. The results show that carbon dioxide fixation can occur without a magnesium ion, and binding with C1 can have a lower barrier (~12 kcal/mol) than with C2 (~23 kcal/mol).

## 1. Introduction

The lifeforms and ecosystems on planet Earth would look completely different in the absence of photosynthesis, which arose around 3 billion years ago. Thanks to its functioning, the atmosphere of the Earth was enriched with oxygen and new means of chemical exchange became possible. When using this element, the efficiency of oxidative exchange reactions reaches 40%, in contrast to 8–10% when using ferrous iron and sulfur. The high energy efficiency of oxygen anabolism has, as a result, become the basis for the organization of complex food chains, in which competition has contributed to the increased sizes of lifeforms. In addition, oxygen is involved in the formation of strong skeletons in animals and lignin in plants, which is a fundamental requirement for the growth and functioning of physically large organisms. In the absence of free O_2_ in the atmosphere, the inhabitants of the Earth would be very limited in size, morphologically rather primitive, and could not have mastered the vast expanses of land.

Due to the extraordinary complexity and multilevel hierarchy of the mechanisms of photosynthesis, it remains largely a poorly understood phenomenon, at least in regard to its unusually low efficiency, which greatly limits the productivity of agricultural plants. In part, nature itself has corrected the previously mastered C3 method of carbon fixation and about 20 million years ago “created” a more efficient C4 cycle. However, it exists only in a small number of genera and is very limited, both ecologically and geographically [1]. Among cultivated cereals, C4 fixation of carbon dioxide is carried out in maize and sorghum. The main grain crops—wheat, rye, barley, and rice—use C3 fixation, and as a result are significantly inferior in terms of yield. To overcome this limitation, research is being carried out across the world to improve the Benson–Bassem–Calvin (BBC) cycle, which is the main way of incorporating CO_2_ in organic metabolism [2], and to explore the possibility of replacing it with other fundamentally different variants of carboxylation reactions during the assimilation of carbon dioxide in plants [3,4,5,6].

The first step in the carboxylation mechanism of ribulose-1,5-bisphosphate (RuBP) catalyzed by Rubisco in the BBC cycle is the formation of enediolate, which can then attach CO_2_ or O_2_ molecules. In the case of the attachment of CO_2_ to enediolate, an intermediate 6-carbon compound is formed, which is named 3-keto-2-carboxy-d-arabinitol 1,5-bisphosphate (3kCABP). In the literature, one can find a number of works devoted to the modeling of the steps of the carboxylation of RuBP by quantum–chemical methods [7,8,9,10], as well as a combination of quantum–chemical and molecular modeling methods [11,12,13,14]. When modeling the RuBP enolization reaction, some authors consider the reaction product to be the 2,3-enediol compound [11] or its deprotonated form, 2,3-enediolate [7,8,9,10,11,12,13,14]. In this study, we assume that 1,2-enol or its deprotonated form can be formed during the enolization step. Herein, we use 2,3-enediol and 1,2-enol to abbreviate the names of key intermediates with the following full names: 2,3,4-trihydroxypent-2-ene-1,5-diyl diphosphate and 2,3,4-trihydroxypent-1-ene-1,5-diyl diphosphate. Since the differences between the two are minor, we also use the generic name 1/2-ene(mono/di)ol.

To determine the kinetics, most authors use an enzyme model in which the 2,3-enediolate forms a complex with the magnesium ion Mg^2+^. In previous studies [7,8,9,12,13] it was shown that the formation of 3kCABP is energetically favorable; when the 2,3-enediolate interacts with the CO_2_ molecule the equilibrium shifts towards the reaction products. Kannappan and Grady point out that Rubisco is a very ineffective enzyme with a low catalytic rate of CO_2_ fixation [7]. Therefore, in this study, the steps of enolization and CO_2_ fixation are modeled without the magnesium ion. We hope that authors studying the influence of Rubisco on the pathway of the carboxylation reaction may be interested in the mechanisms of enolization and CO_2_ fixation when the oxygen atoms of RuBP cannot bind to the magnesium ion.

These modeling features were chosen since in a previously published work [6] we considered the possibility of increasing the efficiency of photosynthesis by changing the enolization pathway of ribulose-1,5-bisphosphate and the attachment of carbon dioxide to the first carbon atom of the RuBP with the formation of 2,6-bisphosphate gluconic acid (BPGA). The carboxylation of RuBP via this pathway immediately leads to the formation of 6-atomic carbohydrates and avoids photorespiration, which theoretically should give at least a 25% increase in plant productivity. Thus, the main objectives of the study are: (1) estimation of the energy barrier for RuBP carboxylation by the first carbon atom; (2) comparison to the barrier of carboxylation in the classical BBC cycle.

## 2. Results

**Figure 1:**
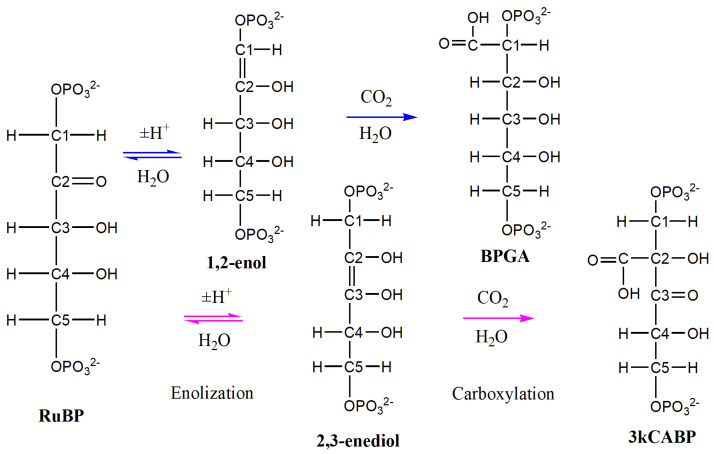
Pathways of enolization and carboxylation reactions via carbon atoms C_1_ (blue) and C_2_ (purple).

In order to reveal the preferred pathways for the fixation of carbon dioxide by the 1/2-ene(mono/di)ol molecules, quantum–chemical calculations of the reagents, their key intermediates and probable products were performed. Figure 1 shows the schematic structures of the main compounds under study. Table 1 shows the energy characteristics of the compounds, as well as the charges on the atoms involved in the formation of the double bonds between the carbons of the 1/2-ene(mono/di)ol molecules. Rows 1–3 of the table lists the main reagents: RuBP, including its two protonated forms with an explicit water molecule, RuBP1^+^⋅H_2_O (a proton attached to a carbonyl oxygen) and RuBP2^+^⋅H_2_O (a proton attached to one of the phosphate oxygens). The key intermediates are listed in rows 4–7. The characteristics of 1/2-ene(mono/di)olate ions are also calculated, since 2,3-enediolate is traditionally used in many enolization and carboxylation reaction schemes. The possible products of carboxylation are listed in rows 8–11. Since BPGA has an increased number of hydrogen atoms compared to 3kCABP, it is difficult to evaluate the energetically favorable carboxylated form after CO_2_ fixation. Therefore, additional quantum–chemical calculations were performed for CABP, which contains the same number of atoms as BPGA (Appendix A).

**Figure A1:**
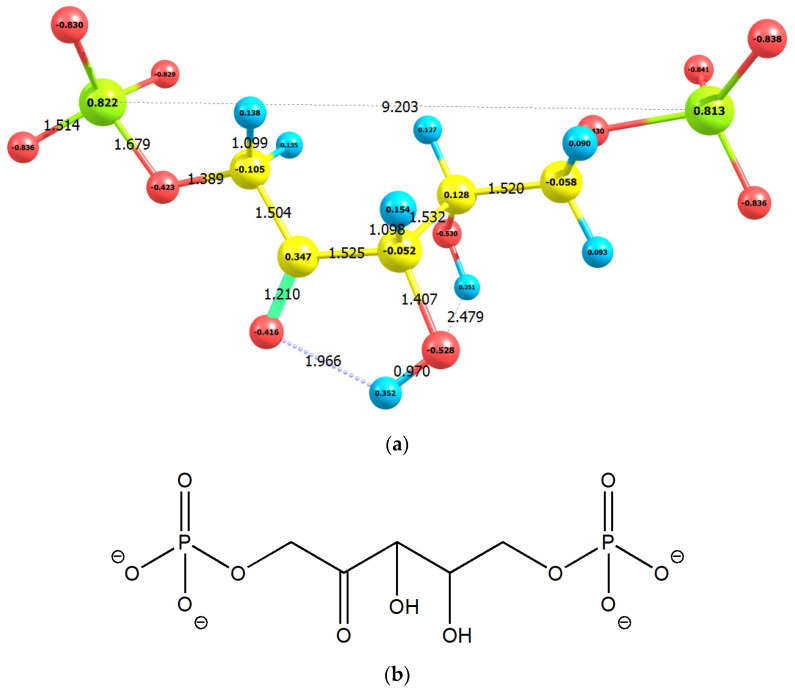
(**a**) The calculated geometric and electronic structure of ribulose-1,5-bisphosphate (RuBP); Q = −4, M = 1, E_0_ = −46,437.91 eV, G_0_ = −46,434.57 eV. Hessian has negative frequencies: −31.54, −7.59 (cm^−1^). (**b**) The scheme of 2,3-dihydroxy-4-oxopentane-1,5-diyl diphosphate.

**Figure A2:**
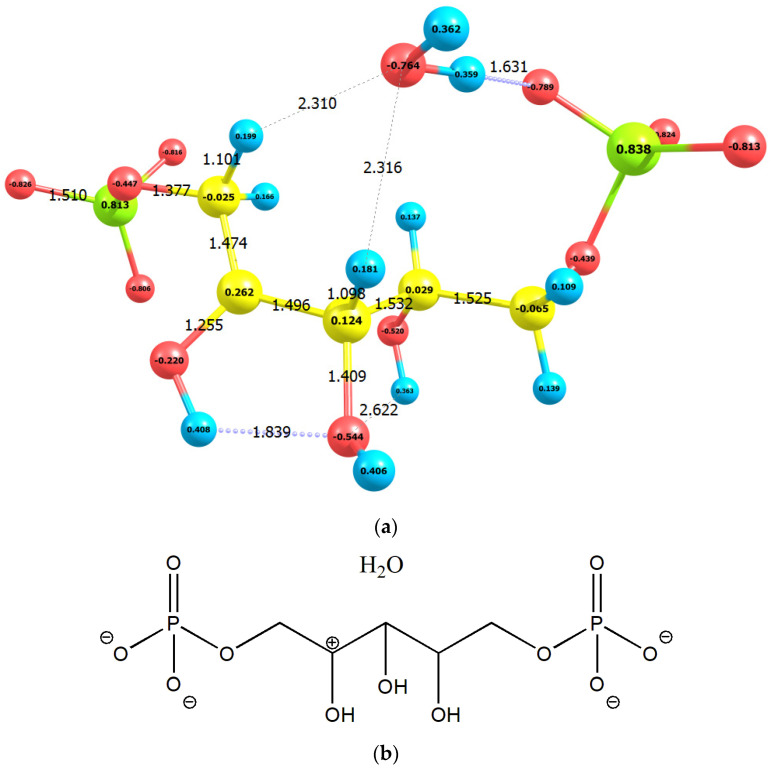
(**a**) The calculated geometric and electronic structure of protonated ribulose-1,5-bisphosphate with single water molecule (RuBP1^+^⋅H_2_O); Q = −3, M = 1, E_0_ = −48,530.12 eV, G_0_ = −48,525.85 eV. Hessian has negative frequencies: −26.56 (cm^−1^). (**b**) The scheme of 2,3,4-trihydroxypentan-2-ylium-1,5-diyl diphosphate.

**Figure A3:**
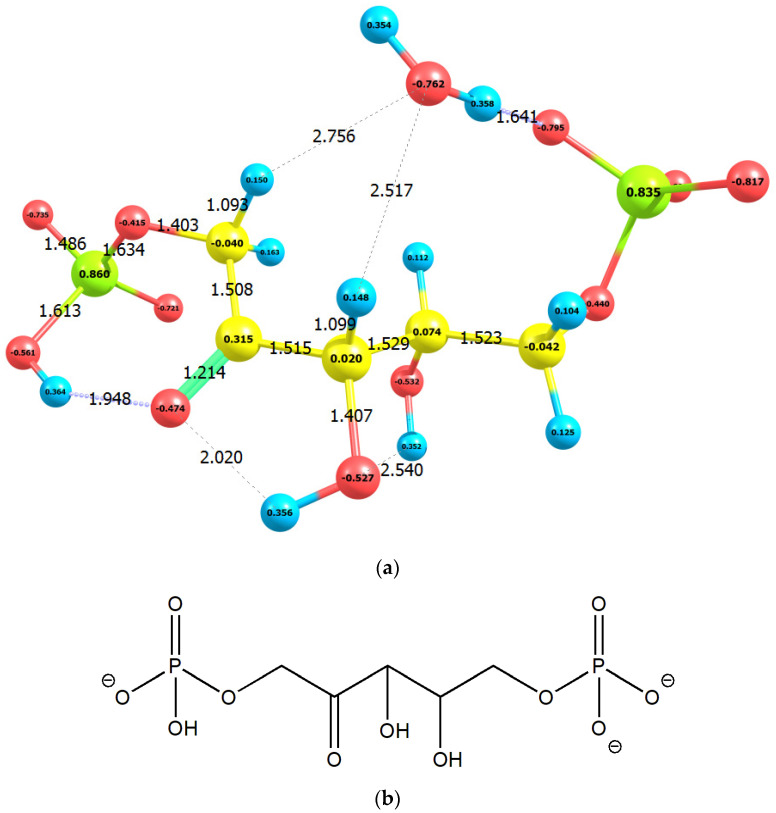
(**a**) The calculated geometric and electronic structure of protonated ribulose-1,5-bisphosphate with single water molecule (RuBP2^+^⋅H_2_O); Q = −3, M = 1, E_0_ = −48,531.77 eV, G_0_ = −48,527.58 eV. Hessian has no negative frequencies. (**b**) The scheme of 5-(hydrogen phosphonatooxy)-2,3-dihydroxy-4-oxopentyl phosphate.

**Figure A4:**
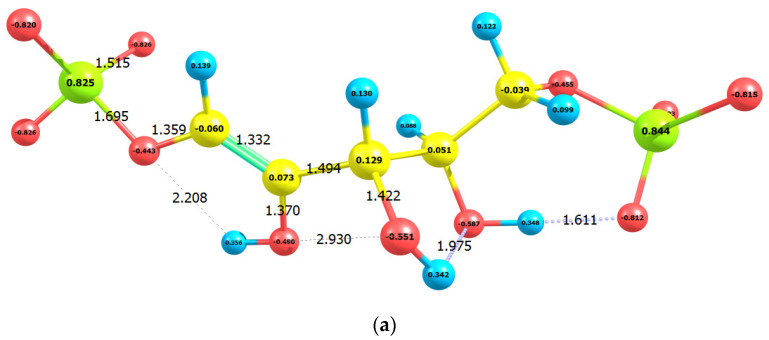

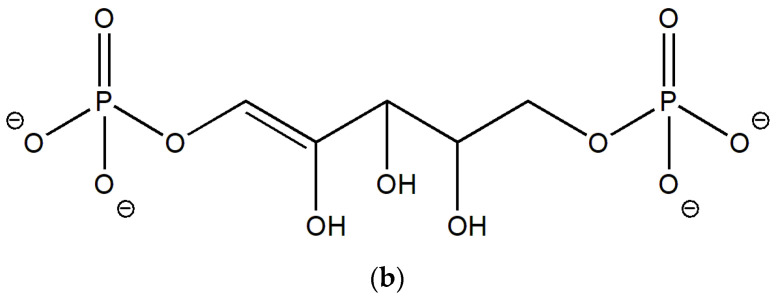
(**a**) The calculated geometric and electronic structure of 1/2-ene(mono/di)ol (1,2-enol); Q = −4, M = 1, E_0_ = −46,437.76 eV, G_0_ = −46,434.44 eV. Hessian has no negative frequencies. (**b**) The scheme of (Z)-2,3,4-trihydroxypent-1-ene-1,5-diyl diphosphate.

**Figure A5:**
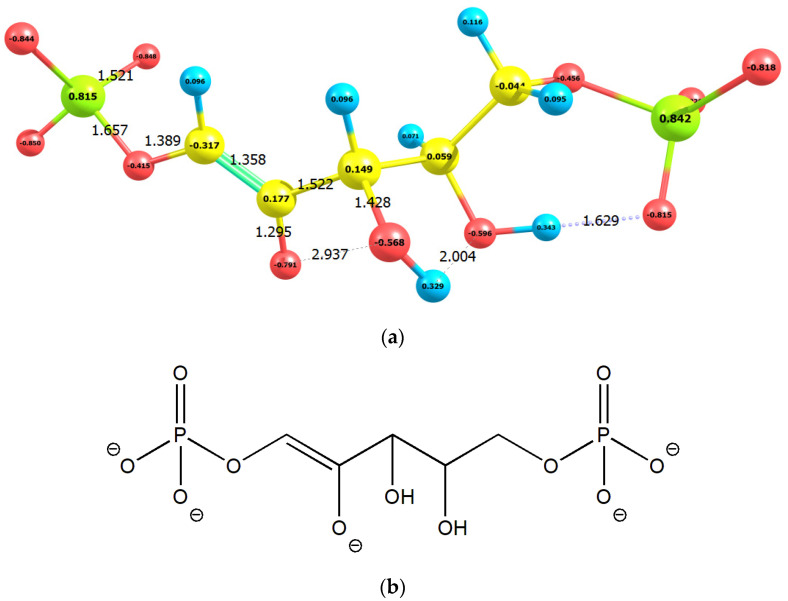
(**a**) The calculated geometric and electronic structure of 1/2-ene(mono/di)olate (1,2-enolate); Q = −5, M = 1, E_0_ = −46,424.13 eV, G_0_ = −46,421.11 eV. Hessian has negative frequencies: −135.86, −98.86 (cm^−1^). (**b**) The scheme of (Z)-3,4-dihydroxy-2-oxidopent-1-ene-1,5-diyl diphosphate.

**Figure A6:**
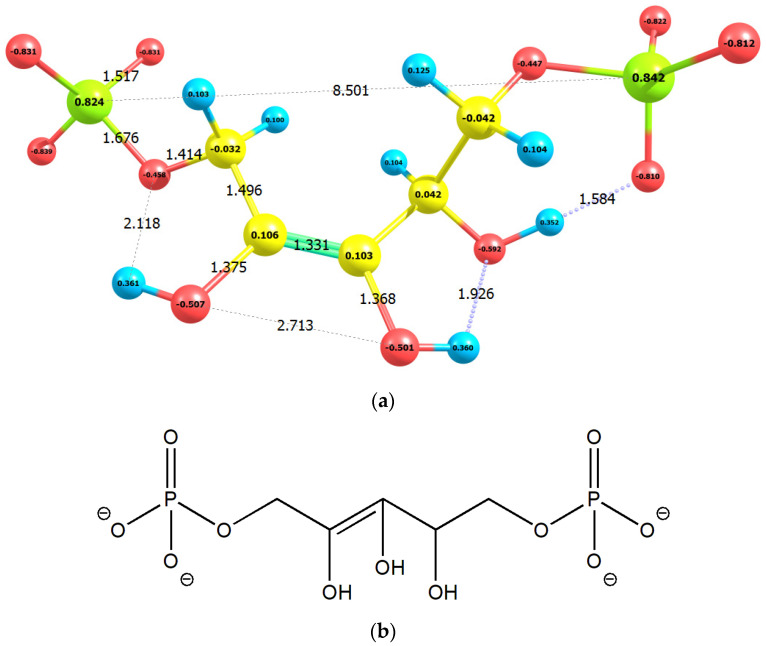
(**a**) The calculated geometric and electronic structure of 1/2-ene(mono/di)ol (2,3-enediol); Q = −4, M = 1, E_0_ = −46,437.74 eV, G_0_ = −46,434.39 eV. Hessian has negative frequencies: −24.51 (cm^−1^). (**b**) The scheme of (Z)-2,3,4-trihydroxypent-2-ene-1,5-diyl diphosphate.

**Figure A7:**
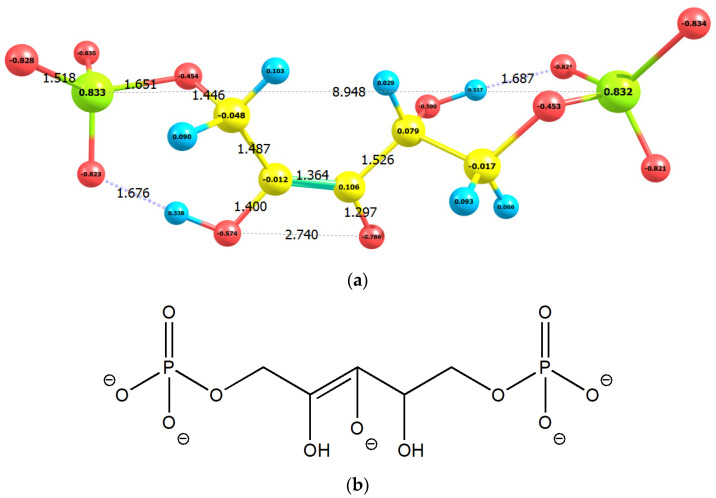
(**a**) The calculated geometric and electronic structure of 1/2-ene(mono/di)olate (2,3-enediolate); Q = −5, M = 1, E_0_ = −46,424.27 eV, G_0_ = −46,421.25 eV. Hessian has negative frequencies: −28.34 (cm^−1^). (**b**) The scheme of (Z)-2,4-dihydroxy-3-oxidopent-2-ene-1,5-diyl diphosphate.

**Figure A8:**
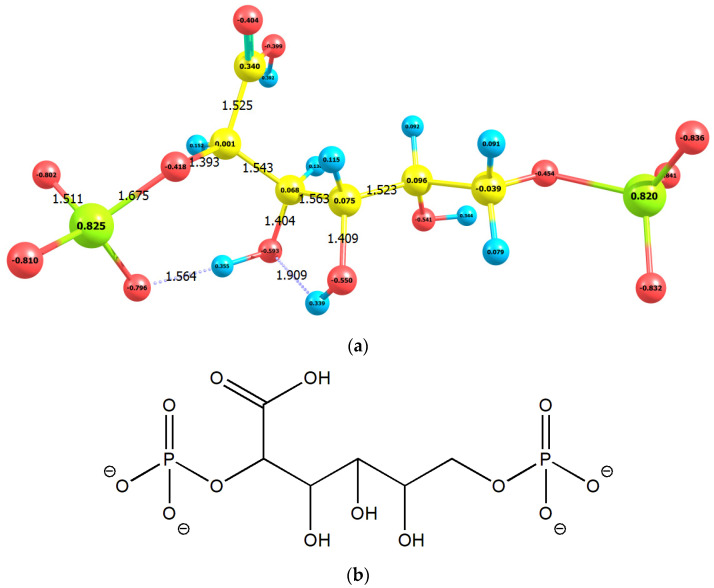
(**a**) The calculated geometric and electronic structure of 2,6-bisphosphate gluconic acid (BPGA); Q = −4, M = 1, E_0_ = −51,603.38 eV, G_0_ = −51,599.04 eV. Hessian has negative frequencies: −33.21 (cm^−1^). (**b**) The scheme of 1-carboxy-2,3,4-trihydroxypentane-1,5-diyl diphosphate.

**Figure A9:**
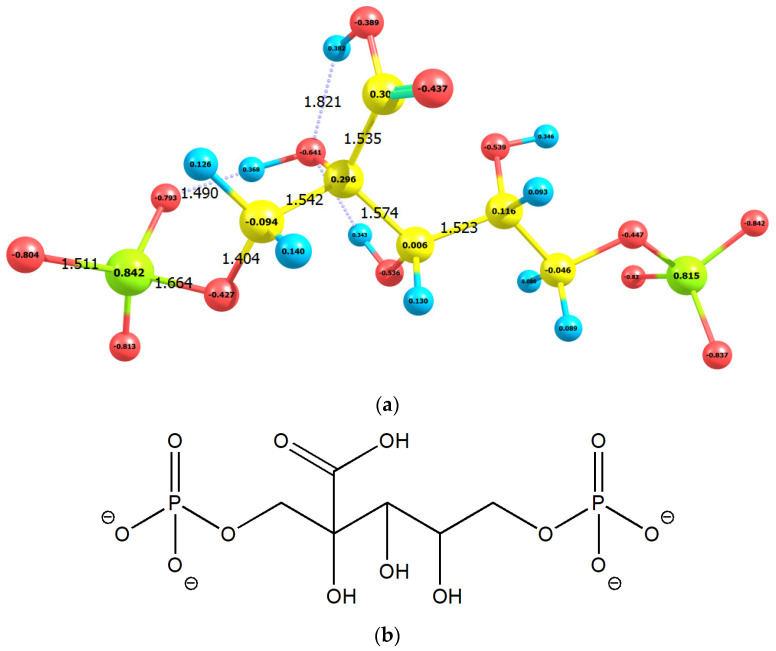
(**a**) The calculated geometric and electronic structure of 2-carboxy-d-arabinitol 1,5-bisphosphate (CABP); Q = −4, M = 1, E_0_ = −51,603.53 eV, G_0_ = −51,599.18 eV. Hessian has negative frequencies: −82.12, −18.16 (cm^−1^). (**b**) The scheme of 2-carboxy-2,3,4-trihydroxypentane-1,5-diyl diphosphate.

**Figure A10:**
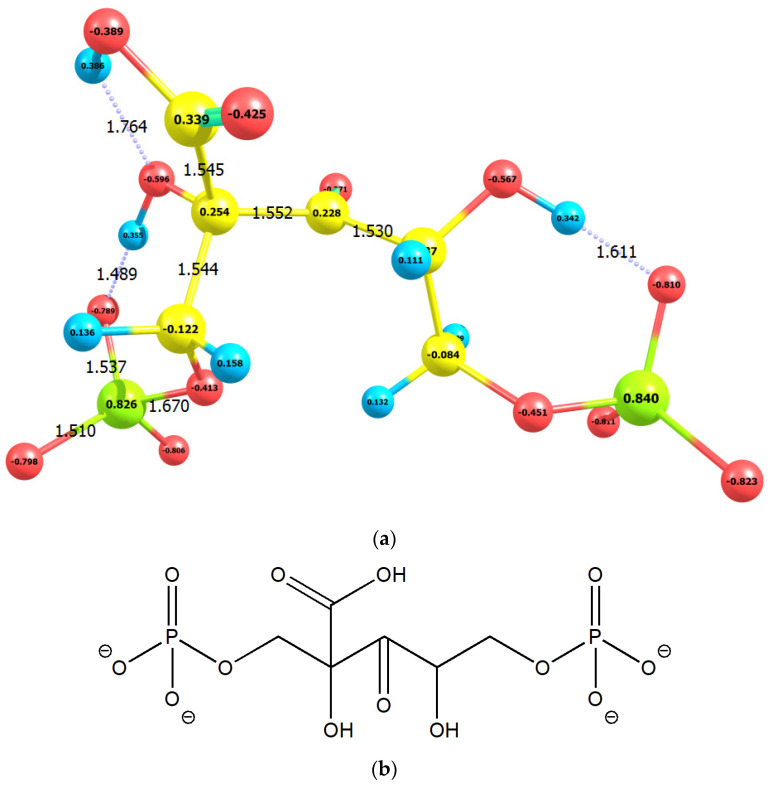
(**a**) The calculated geometric and electronic structure of 3-keto-2-carboxy-d-arabinitol 1,5-bisphosphate (3kCABP); Q = −4, M = 1, E_0_ = −51,570.72 eV, G_0_ = −51,567.08 eV. Hessian has no negative frequencies. (**b**) The scheme of 2-carboxy-2,4-dihydroxy-3-oxopentane-1,5-diyl diphosphate.

**Figure A11:**
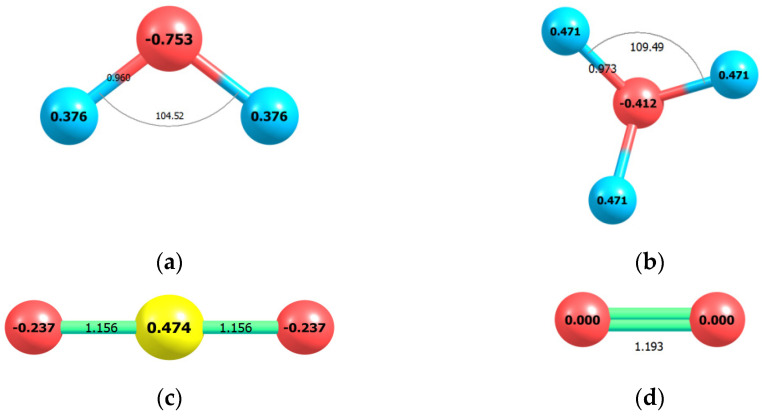
The calculated geometric and electronic structures: (**a**) H_2_O, Q = 0, M = 1, E_0_ = −2080.34 eV, G_0_ = −2080.25 eV; (**b**) H_3_O^+^, Q = 1, M = 1, E_0_ = −2091.54 eV, G_0_ = −2091.10 eV; (**c**) CO_2_, Q = 0, M = 1, E_0_ = −5132.52 eV, G_0_ = −5132.76 eV; (**d**) O_2_, Q = 0, M = 3, E_0_ = −4091.14 eV, G_0_ = −4091.58 eV.

The calculated geometric and electronic structures of all compounds included in Table 1 are detailed in Appendix A (Figure A1, Figure A2, Figure A3, Figure A4, Figure A5, Figure A6, Figure A7, Figure A8, Figure A9, Figure A10 and Figure A11). The molecules RuBP, 1/2-ene(mono/di)ol, BPGA and CABP have a linear structure. The carbon atoms C_1_-C_5_ form an extended chain. The distance between the phosphorus of the OPO_3_ groups is in the range of 8.5–9.3 Å. The carbon chain of the 3kCABP molecule is more compact, and the molecule is globular in shape. The distance between phosphorus atoms is 7.3 Å.

Analyzing the bond lengths of the molecules shown in Figure A1, Figure A2, Figure A3, Figure A4, Figure A5, Figure A6, Figure A7, Figure A8, Figure A9 and Figure A10, we see that strong hydrogen bonds are formed between the hydrogen and oxygen atoms of the hydroxyl groups. The oxygen atoms of the OPO_3_ groups are also involved in the formation of hydrogen bonds. Due to the formation of additional hydrogen bonds, the total energy of the molecule can be slightly reduced. Consequently, the globular structure of molecules is more energetically favorable, which makes it possible to explain the appearance of negative frequencies in the Hessian for some linear molecules. Table 1 shows that the charges on carbon atoms can change significantly due to protonation of the RuBP molecule, deprotonation of 1/2-ene(mono/di)ol, and the transformation of CABP molecule into its 3-keto form.

### 2.1. Enolization

According to the scheme presented in ref. [11] the conversion of RuBP to 2,3-enediol requires a proton exchange between carbon C_3_ and the carbonyl group. The authors consider that the enzyme catalyzes enolization with a specific active-site residue, which acts as a base that removes protons from the C_3_ atom, and afterward an acid protonates the carbonyl oxygen. We suppose that the bonding of the proton with the carbonyl oxygen RuBP can contribute to the proton detachment from the C_3_ carbon, since the charge on the C_3_ atom becomes positive (Figure A2). A decrease in the C_1_–C_2_ and C_2_–C_3_ bond lengths in RuBP1^+^ can lead to protonated 1/2-ene(mono/di)ol with C_1_=C_2_ or C_2_=C_3_ double bonds.

The protonated form: RuBP2^+^ (Figure A3) is obtained by calculating RuBP1^+^ when the proton is oriented towards the OPO_3_ group. Geometrical optimization unexpectedly results in proton transfer from the carbonyl oxygen to the nearest oxygen of the OPO_3_ group. Indeed, RuBP2^+^ has a lower total energy than RuBP1^+^ and the difference is significant, at 1.65 eV. However, no decrease in the lengths of the C_1_–C_2_ and C_2_–C_3_ bonds is observed. The electronegativity of C_1_ and C_3_ atoms decreases, but not as much as upon protonation of carbonyl oxygen in RuBP1^+^.

**Figure 2:**
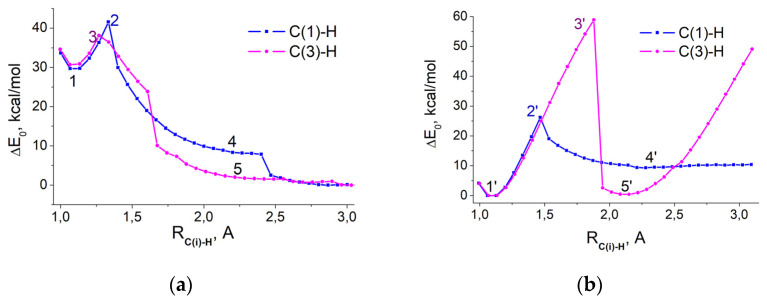
The energy relaxed surface scans of proton detachment from C_1_ (blue) and C_3_ (purple) carbons in: (**a**) RuBP1^+^⋅H_2_O; E_0_ = −48,530.12 eV (−1,119,104.57 kcal/mol). (**b**) RuBP2^+^⋅H_2_O; E_0_ = −48,531.77 eV (−1,119,142.62 kcal/mol).

To estimate the probability of proton detachment from C_1_ and C_3_ atoms, let us calculate the relaxed surface scans of the total energies along the reaction coordinates R_C(1)-H_ and R_C(3)-H_. Both protonated forms RuBP1^+^ and RuBP2^+^ are complemented by a single water molecule (Figure A2 and Figure A3), which can act as a proton acceptor and a bridge to the transport of protons from carbon C_1_ or C_3_ to the oxygen of the OPO_3_ group. In each model reaction, the C-H bond length sequentially increases by 0.1 A. The reaction coordinate has a fixed value, while all other geometric parameters are optimized to calculate the dependence of the total energy on the reaction coordinate. Figure 2 shows the total energies of model reactions with respect to the total energy, E_0_, of the initial configurations presented in Figure A2 and Figure A3.

**Figure 3:**
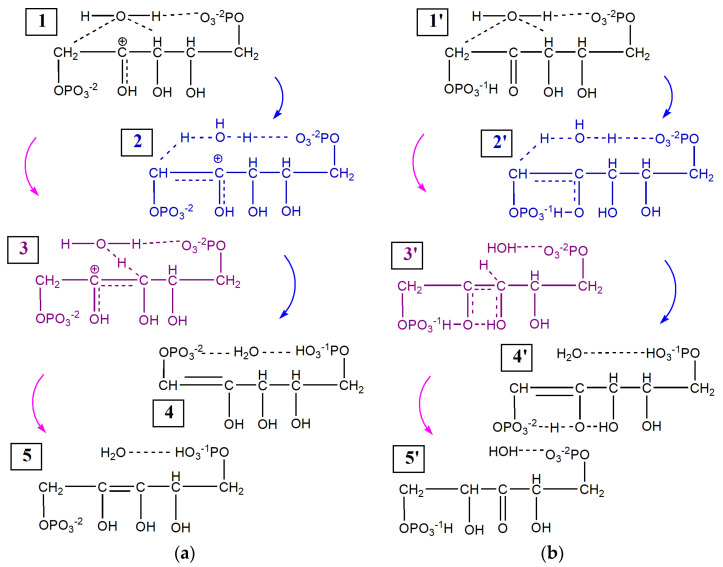
The scheme of enolization via proton detachment from C_1_ (blue) and C_3_ (purple) carbons in: (**a**) RuBP1^+^⋅H_2_O; (**b**) RuBP2^+^⋅H_2_O. The initial states are (1) and (1′). The states corresponding to the top of the barrier (2), (3) and (2′), (3′) are colored. The products of enolization are as follows: (Z)-5-(hydrogen phosphonatooxy)-2,3,4-trihydroxypent-1-enyl phosphate—states (4) and (4′), (Z)-5-(hydrogen phosphonatooxy)-2,3,4-trihydroxypent-2-enyl phosphate—tate (5). The product of tauto-merization is 5-(hydrogen phosphonatooxy)-2,4-dihydroxy-3-oxopentyl phosphate— state (5′).

To remove a proton from carbon atoms C_1_ or C_3_, it is necessary to overcome a barrier of about 45 kcal/mol (Figure 2a). Acting as a bridge, the water molecule can accept the detached proton, while the water proton is transferred to the oxygen of the OPO_3_ group, resulting in the formation of the protonated 1,2-enol^+^ (states 4 and 4′ in Figure 3) or 2,3-enediol^+^ (state 5 in Figure 3). These pathways are represented in Figure 3 by transformations 1 → 2 → 4, 1 → 3 → 5 and 1′ → 2′ → 4′. In the case of 1′ → 3′ → 5′, the proton detached from carbon C_3_ is transferred to carbon C_2_, which does not lead to the protonated form of 1/2-ene(mono/di)ol. It should be noted for the 1′ → 2′ → 4′ pathway, that the proton of the OPO_3_^−1^H group can be transferred to the carbonyl oxygen, while the proton of water is transferred to the opposite OPO_3_ group.

Figure 3a shows that the initial state (1), RuBP1^+^⋅H_2_O, has a higher energy than the configurations of products (4) and (5). In this case, in order to remove a proton from carbon C_1_ or C_3_, it is necessary to overcome the barrier of 8–11 kcal/mol. However, to obtain an advantageous initial configuration, approximately an additional 30 kcal/mol of energy is required. Provided that protons can easily attach and detach from OPO_3_ groups, we can assume that both reaction pathways lead to the equally likely formation of both 1,2-enol^+^ and 2,3-enediol^+^.

In the case of the initial state (1′), RuBP2^+^⋅H_2_O, only one pathway 1′ → 2′ → 4′ leads to the formation of protonated 1,2-enol^+^ (4′). The barrier is 28 kcal/mol (Figure 2b), which is much less than the 48 kcal/mol observed for the 1 → 2 → 4 transformations (Figure 2a). To provide the tautomerization pathway, 1′ → 3′ → 5′, it is necessary to overcome the 60 kcal/mol barrier and to reach a distance, R_C(3)-H_ ~ 2 Å, whereas enolization occurs at distances not exceeding 1.5 Å.

### 2.2. CO_2_ Fixation

Since the typical carboxylation reaction models are based on the interaction of CO_2_ with 2,3-enediolate [7,8,9,10,11,12,13,14], let us compare the activities of 1,2-enol and 1,2-enolate with 2,3-enediol and 2,3-enediolate, respectively. The activities of the molecules 1/2-ene(mono/di)ol and 1/2-ene(mono/di)olate can be estimated from the quantum–chemical analysis of the charges on the C_1_-C_3_ carbon atoms and the electronic structure of the boundary molecular orbitals (rows 4–7 in Table 1).

The electronic and geometric structures of 1/2-ene(mono/di)ol molecules are shown in Figure A4 and Figure A6, and their deprotonated forms are shown in Figure A5 and Figure A7. In the case of 1,2-enol, deprotonation of the OH group attached to the C_1_ carbon leads to an increase in the negative charge q_C1_ by a factor of 6 (rows 4, 5 in Table 1). In the case of 2,3-enediol, deprotonation of the OH group attached to the C_2_ carbon causes a change in the sign of the charge q_C(2)_ from positive to negative (rows 6, 7 in Table 1). An increase in the electronegativity of active centers promotes their interaction with the positively charged carbon atom of the CO_2_ molecule. In addition, the deprotonation of 1/2-ene(mono/di)ol leads to a slight lengthening of the double bond between carbon atoms: C_1_=C_2_ increases by 0.026 Å in 1,2-enolate and C_2_=C_3_ increases by 0.033 Å in 2,3-enediolate.

The 1/2-ene(mono/di)ol molecules have insignificant differences in electronic structure, which is expressed in the similar values of the electronic charges on the C_1_-C_5_ carbon atoms and close values of the total energies and energies of the boundary molecular orbitals (rows 4 and 6 in Table 1). The main contribution to HOMO of 1,2-enol is made by the following AOs: p_x_(C_1_) = 0.17, p_x_(C_2_) = 0.19, p_z_(C_1_) = 0.02, p_z_(C_2_) = 0.02. The main contribution to the HOMO of 2,3-enediol is made by the following AOs: p_z_(C_2_) = 0.11, p_z_(C_3_) = 0.10, p_y_(C_2_) = 0.09, p_y_(C_3_) = 0.09. In each case, the main contribution is made by the π-orbitals participating in the formation of the double bond.

After deprotonation of the 1/2-ene(mono/di)ol molecules, the main contributions of AOs to HOMO undergo several significant changes. The AO contribution of C_1_ carbon to the HOMO of 1,2-enolate becomes dominant: p_x_(C_1_) = 0.29, p_x_(C_2_) = 0.08, p_z_(C_1_) = 0.01, p_z_(C_2_) = 0.02, while the AO contribution of C_2_ carbon to HOMO predominates in 2,3-enediolate: p_z_(C_2_) = 0.17, p_z_(C_3_) = 0.06, p_y_(C_2_) = 0.1, p_y_(C_3_) = 0.04. Electrophilic interaction is preferably with the C_1_ atom in the 1,2-enolate and with the C_2_ atom in the 2,3-enediolate. The HOMO energy of 1,2-enediolate becomes 0.14 eV lower than the HOMO energy of 2,3-enediolate. Thus, the interaction energy between the electrophilic agent (CO_2_) and 2,3-enediolate are lower than those of 1,2-enolate, which makes it possible to provide a better affinity for carbon dioxide and to obtain an energetically more favorable product of the carboxylation reaction.

Carboxylation via carbon C_1_ leads to the formation of the product 2,6-bisphosphogluconic acid (BPGA, Figure A8) during the reaction of 1,2-enol(ate) with carbon dioxide according to the scheme shown in Figure 1 (blue pathway). In the classic scheme, 2,3-enediol(ate) reacts with carbon dioxide via carbon C_2_ as shown in Figure 1 (purple pathway), resulting in the well-known 3-keto-2-carboxy-d-arabinitol -1,5-bisphosphate (3kCABP, Figure A10). Further, the 3-keto compound decomposes at the C_2_–C_3_ bond into two 3-phosphoglycerate molecules. BPGA decomposition requires further study to explain its incorporation into the metabolic pathway.

To compare the energy characteristics of the carboxylation products for both reaction schemes, the 2-carboxy-d-arabinitol 1,5-bisphosphate (CABP, Figure A9) are calculated. CABP contains the same number of hydrogen atoms as BPGA. Calculations show that the formation of CABP or its 3-keto form is energetically more favorable than the formation of BPGA. The difference in total energies is 0.15 eV, and the difference in Gibbs free energy is 0.14 eV (3.2 kcal/mol). This result shows that 3kCABP is thermodynamically more likely than BPGA. On the other hand, 3kCABP can be quite stable, which can make it difficult to decompose into two 3-phosphoglycerate molecules. Indeed, early attempts to simulate scission of the C_2_–C_3_ bond demonstrate that the energies of the final products can be higher than the energy of intermediate reagents [7,13].

**Figure 4:**
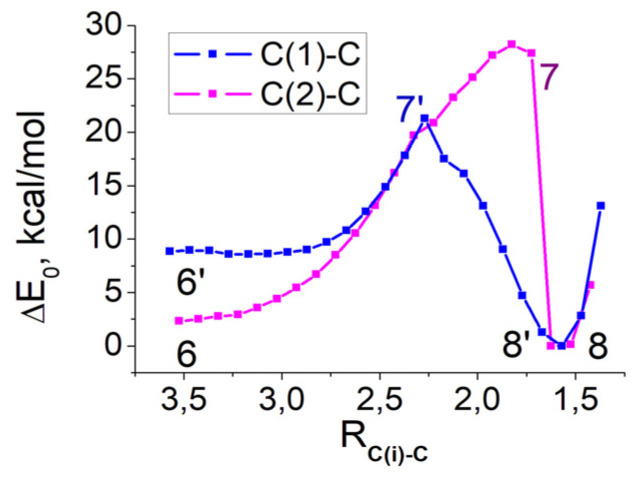
The energy relaxed surface scans of carboxilation via C_1_ (blue) and C_2_ (purple) carbons, E_f_ = −53,664.65 eV (−1,237,534 kcal/mol).

**Figure 5:**
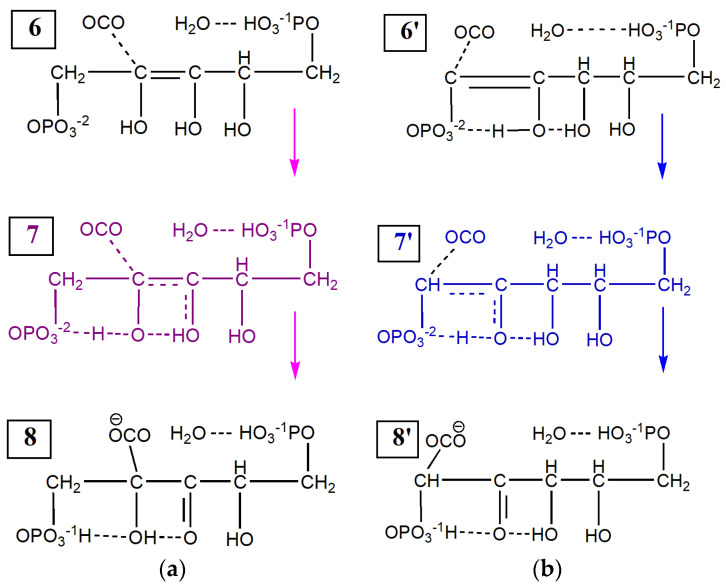
The scheme of carboxylation via C_1_ (blue) and C_2_ (purple) carbons in: (**a**) 1,2-enol^+^⋅H_2_O; (**b**) 2,3-enediol^+^⋅H_2_O. The initial states (6) and (6′) are preliminarily calculated at the stages of enolization. Their optimized geometric structures are shown in Figure A12 and Figure A13, respectively. The states corresponding to the top of the barrier (7) and (7′) are colored. The products of carboxylation are as follows: 5-(hydrogen phosphonatooxy)-2-((hydrogen phosphonatooxy)methyl)-2,4-dihydroxy-3-oxopentanoate—the state (8) excluding water and 2,6-bis(hydrogen phosphonatooxy)-4,5-dihydroxy-3-oxohexanoate—the state (8′) excluding water.

**Figure A12:**
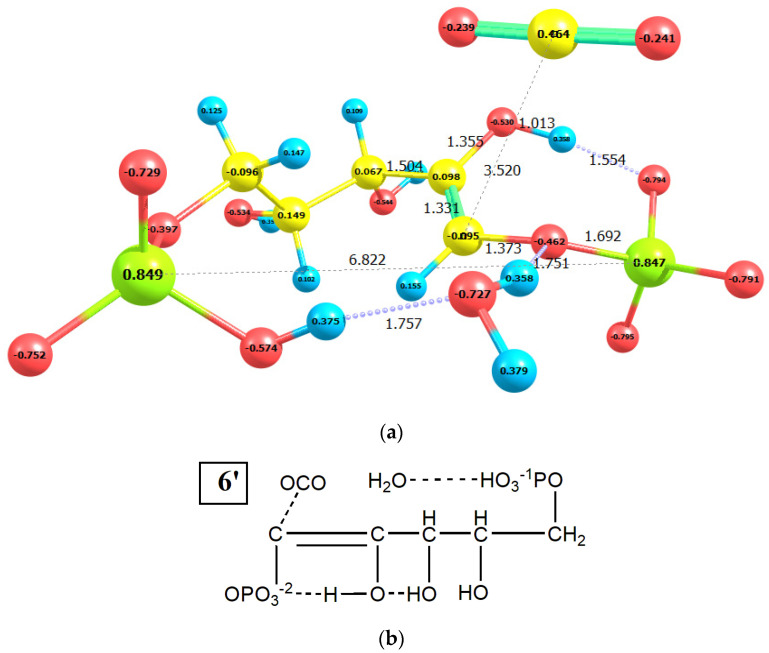
(**a**) The calculated geometric and electronic structures of the initial state of carboxylation in the C1 direction; Q = −3, M = 1, E_0_ = −53,664.08 eV, G_0_ = −53,659.65 eV. Hessian has no negative frequencies. (**b**) The scheme of state (6′) is pictured in Figure 5b.

**Figure A13:**
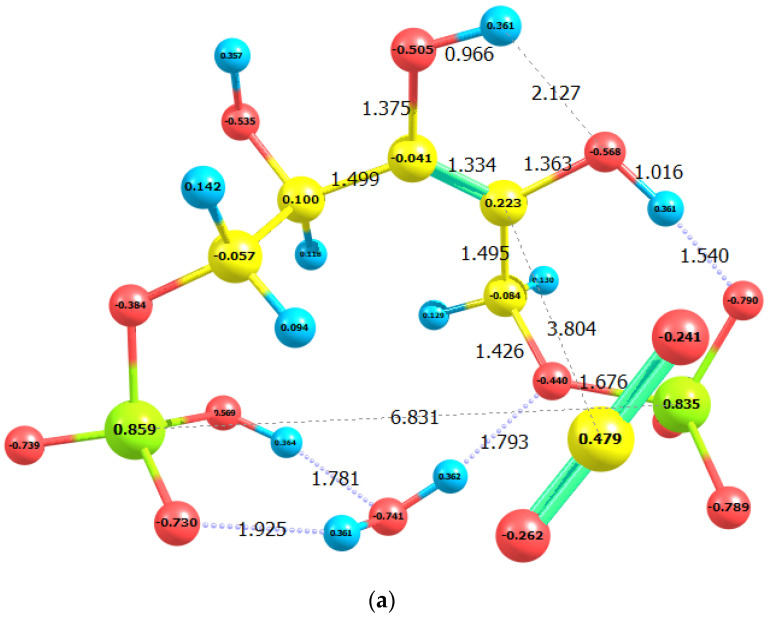

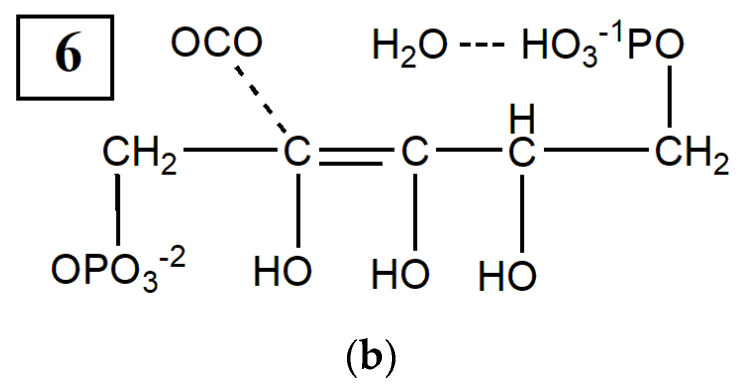
(**a**) The calculated geometric and electronic structure of initial state of carboxylation in C2 direction; Q = −3, M = 1, E_0_ = −53,664.37 eV, G_0_ = −53,659.83 eV. Hessian has no negative frequencies. (**b**) The scheme of state (6) is pictured in Figure 5a.

Using the enolization products of 1,2-enol^+^⋅H_2_O (state 4′) and 2,3-enediol^+^⋅H_2_O (state 5) together with carbon dioxide as the initial states (Figure A12 and Figure A13), we calculate the relaxed surface scans along the reaction coordinates R_C(1)-C_ and R_C(2)-C_. In each model reaction, the length of the C*_i_*–C bond between 1/2 carbons of the protonated 1/2-ene(mono/di)ol and carbon of CO_2_ is consequently decreased by 0.1 Å. The reaction coordinate has a fixed value, and all other geometric parameters are optimized to calculate the dependence of the total energy on the reaction coordinate. Figure 4 shows the difference between the total energies calculated for each value of the reaction coordinate with respect to the total energy of the final configuration having the lowest energy, E_f_. Figure 5 shows two carboxylation schemes via carbon atoms C_1_ (blue pathway) and C_2_ (purple pathway).

Both the C_1_ and C_2_ pathways of CO_2_ fixation make it possible to obtain a six-carbon compound. Proton transfer between the hydroxyl groups and the oxygen of the OPO_3_^−2^ group plays a crucial role in the fixation of CO_2_ and the formation of C_2_=O or C_3_=O double bonds. The initial state 6′ has a higher total energy value than state 6 because fewer hydrogen bonds are formed here (compare Figure A12 and Figure A13). When state 6 is considered as the initial one, the attachment of CO_2_ to the C_2_ atom and further the formation of the C_3_=O double bond requires the larger consumption of energy due to the simultaneous transfer of two protons, as well as the greater destruction of hydrogen bonds. Thus, the energy barrier of CO_2_ binding to carbon C_1_ (13 kcal/mol) is lower than to carbon C_2_ (27 kcal/mol).

## 3. Discussion

The quantum–chemical modeling of enolization and carboxylation reactions performed in this work differs from the reaction pathways presented in Figure 1. The main difference lies in the choice of active forms of reagents and intermediate compounds that we use to calculate the reaction barriers and assess the energy properties of the compounds used in the literature. To compare both approaches, it is possible to estimate the thermodynamic properties of the reagents and products included in Figure 1, based on the results of calculating the Gibbs free energy.

Let us compare the Gibbs free energies of RuBP(1/2)^+^∙H_2_O and RuBP together with H_3_O^+^ combined into a common system:

G_0_(RuBP + H_3_O^+^) = −48,525.67 eV. (1)

The Gibbs free energy in Equation (1) is calculated as the summary energy of the molecules in brackets. A minor difference (0.18 eV) is observed between the energy of RuBP1^+^∙H_2_O and the sum of the energies of its constituents, which should be a consequence of a decrease in the Gibbs free energy of RuBP1^+^∙H_2_O due to the formation of additional hydrogen bonds. On the other hand, a larger difference (1.91 eV) is observed for RuBP2^+^∙H_2_O, which points to the advantage of direct protonation of the RuBP diphosphate oxygen.

The next step is to compare the energies of isolated molecules and bound systems participating in the fixation of CO_2_ as intermediate states. We calculate the Gibbs free energy, G_0_(1/2), of the system including 1/2-ene(mono/di)ol (Figure 1) along with CO_2_ and H_3_O^+^ to compare them with G_0_(6′/6) of the systems being the initial states in the model reaction of the carboxylation (Figure A12 and Figure A13):

G_0_(1) = −53,658.50 eV. G_0_(6′) = −53,659.65 eV. (2)

G_0_(2) = −53,658.25 eV. G_0_(6) = −53,659.83 eV. (3)

The difference between G_0_(1/2) and G_0_(6′/6) is about 1.5 eV, which corresponds to the first result observed for RuBP2^+^∙H_2_O. This confirms the high probability of the reaction with protonated diphosphate oxygen.

Finally, we compare the products shown in Figure 1, adding simple molecules, with the products obtained in the model reaction to equalize them in terms of the number of hydrogen and oxygen atoms. Applying the alignment procedure to the products obtained via pathway C_1_, we calculate the Gibbs free energies:

G_0_(BPGA + H_3_O^+^) = −53,690.14 eV. G_0_(8′ + H_2_O − ½O_2_) = −53,699.01 eV. (4)

To align the oxygen atoms in the left and right parts of Equation (4), the one oxygen should be excluded from the right part. The Gibbs free energy in the left part is the summary energy of the molecules in brackets. To compare the Gibbs free energies of both products, the sum of the energies of the state (8′) and H_2_O in the right part are reduced by half ^3^O_2_ energy, as indicated in the caption to Figure A10d.

Applying the alignment procedure to the products obtained via the C_2_ pathway, we calculate the Gibbs free energies:

G_0_(3kCABP + H_3_O^+^) = −53,658.18 eV. G_0_(8) = −53,664.55 eV. (5)

The Gibbs free energy in the left part is the summary energy of molecules in brackets. Although the BPGA and 3kCABP compounds in the left parts of Equations (4) and (5) are considered as final products, their energies are higher than those in the right parts. Both pathways give differences of the order of 6–9 eV between the products shown in Figure 1 and the products having the minimum of curves in Figure 5. Since two protons are attached to both phosphate groups, the Gibbs free energies of the structures (8/8′) are greatly reduced. The formation of multiple hydrogen bonds in these model systems can also reduce energy.

Calculations of the Gibbs free energies of intermediate compounds and products make it possible to compare their concentrations for each pathway of carboxylation. We consider that the concentrations of 1,2-enol and 2,3-enediol can be in equilibrium, since their Gibbs free energies differ insignificantly. This difference can be compensated for by the reconfiguration of molecules and the rearrangement of hydrogen bonds in the course of thermal fluctuations. Taking into account the lower reaction barrier via the C_1_ pathway and the same Gibbs free energies for (8/8′) systems, we believe that the CO_2_ binding to the C_1_ atom is more likely.

Quantum–chemical calculations of the energy barriers of the proton detachment from carbon C_1_ or C_3_ show that modeling can be successfully carried out for a protonated RuBP molecule (Figure 3). In this case it is possible to calculate the reaction barrier considering only one reaction coordinate; however, the exchange of protons usually occurs simultaneously, so a more correct simulation is to calculate the relaxed surface scan, which includes two or more reaction coordinates. So the reaction barriers for some pathways can be significantly lower, and the disruption observed for the stage 3′ → 5′ in Figure 3b can be avoided. However, we are convinced that the enolization barriers calculated using the same procedures and conditions for both C_1_ and C_3_ pathways are an acceptable means for comparing the probabilities of 1/2-ene(mono/di)ol^+^ formation.

The additional proton in the RuBP system explains the important role of OPO_3_ groups; the proton has the advantage of attaching to one of the oxygen atoms of the diphosphate groups. A similar result was found in ref. [9], obtained by the authors in the course of modeling the reaction of carboxylation—the intermediate (Figure 1 in ref. [9]) contains the OPO_3_H^−^ group. In our work, it is shown that the proton detachment from carbon C_1_ or C_3_ can be accompanied by protonation of the OPO_3_ group. CO_2_ binding also promotes proton transfer to the OPO_3_ group. Thus, at least two protons are needed to proceed the carboxylation reaction. The first proton activates RuBP to then produce the intermediate 1/2-ene(mono/di)ol^+^. The transfer of the second proton to another OPO_3_ group facilitates the attachment of CO_2_.

We understand that the fixation of carbon dioxide in plants is a complex process in which many compounds are involved, in addition to the Rubisco enzyme having magnesium ions (Mg^2+^) which provide enzymatic activity. Typically, modeling involves many auxiliary compounds in order to initiate a proton migration and to provide a convenient configuration of RuBP with Mg. A water molecule coordinated with Mg is a bridge between CO_2_ and Mg [7,13,14], an unprotonated HIS24 residue is able to remove the O3 proton from RuBP [13], a GLU60 with an accepted proton is an initiator for the formation of Grotthuss chains. They play a special role in the mechanisms of the enolization and carboxylation reactions [9].

To evaluate the effect of the magnesium ion from the opposite point of view, we use a simple model without Mg^2+^ and auxiliary compounds, except for the water bridge. Activated RuBPs with two different protonated sites—O2 and OPO_3_ are used as the initial states of enolization. The formation of 1,2-enol with a protonated OPO_3_ group occurs only along the C_1_ pathway, overcoming the minimum barrier of 28 kcal/mol. Since the oxygen atoms O2 and O3 are not bonded to Mg, the C_3_ pathway leads to the transfer of a proton from O3 to O2 without the formation of protonated 2,3-enediol. Probably, the interaction of both oxygen atoms with Mg can help to avoid proton transfer between them in RuBP and reduce the barrier of the enolization stage.

Using protonated 1,2-enol, we show that binding of CO_2_ to C_1_ can overcome the barrier of 10–20 kcal/mol. The experimentally observed barriers for the enolization and carboxylation stages are about 15 kcal/mol [9]. Theoretical research carried out in ref. [9] demonstrated good agreement between the calculated and experimental energies of enolization and carboxylation using the Kohn–Sham density functional theory at the B3LYP/6-31G* and B3LYPgCP-D3 levels. This prompted us to choose the WB97X-D3 functional, which are efficient DFT-D3 corrected variants.

Modeling the binding of CO_2_ to C_2_ without taking into account the interaction with Mg shows that the formation of a six-carbon compound is possible with the simultaneous transfer of a proton from O2 to OPO_3_ and from O3 to O2. The barrier in this case is about 25 kcal/mol. However, the interaction of O2 and O3 with the magnesium ion may not require proton transfer from O3 to O2. The reaction can proceed in a classical way.

To clarify the probabilities of these processes, additional studies on carboxylation are required, taking into account Mg and auxiliary compounds, as well as two enolization pathways via the C_1_ and C_3_ atoms. On the other hand, we hope that the study of the mechanisms of enolization and carboxylation presented in this work will help studies of artificial systems for fixing carbon dioxide without the active influence of Rubisco components.

The presented theoretical study is only the first step towards understanding the phenomenon of carboxylation with the participation of 1,2-enol. While this allows probabilistic estimates to be made, experimental studies are undoubtedly required to test this hypothesis.

## 4. Materials and Methods

Quantum–chemical calculations were carried out using the restricted Kohn–Sham DFT process with the WB97X-D3 [15,16,17] functional and the RIJCOSX [18] approximation. The wave function was calculated using the def2-TZVP [19,20] basis set, while the self-consistency procedure was performed using the TightSCF criterion. The DFT grid was set to GRID5 for all def2-TZVP calculations. Before optimization, the initial shape of all biomolecules was set to linear. The convergence criterion for geometry optimization was chosen as TIGHTOPT. No symmetry restrictions were applied in the calculations. The geometric and electronic properties of the optimized structures were obtained using LR-CPCM [21,22,23] to account for the implicit water solvent, and in some cases a single water molecule was added explicitly. The Hessian was calculated to test negative frequencies. The relaxed surface scan was performed to calculate the reaction barriers. The ORCA 4.2.1 [24] software package was used for all calculations. ChemCraft [25] software was used for the visualization of geometric structures.

## Figures and Tables

**Table 1:** Electronic and energy characteristics of the studied molecules. The total charge of the molecule, Q; multiplicity, M; charges on atoms, q_C1_, q_C2_, q_C3_; total energy, E_0_ (eV); Gibbs free energy, G_0_ (eV); energy of the highest occupied molecular orbital, E_HOMO_ (eV); energy of the lowest unoccupied molecular orbital, E_LUMO_ (eV).

No	Molecule	Q	M	q_C1_	q_C2_	q_C3_	E_0_	G_0_	E_HOMO_	E_LUMO_
1	RuBP	−4	1	−0.105	0.347	−0.052	−46,437.91	−46,434.57	−8.08	1.55
2	RuBP1^+^⋅H_2_O	−3	1	−0.025	0.262	0.124	−48,530.12	−48,525.85	−8.27	−0.16
3	RuBP2^+^⋅H_2_O	−3	1	−0.040	0.315	0.020	−48,531.77	−48,527.58	−8.31	1.28
4	1,2-enol	−4	1	−0.060	0.073	0.129	−46,437.76	−46,434.44	−7.64	3.43
5	1,2-enolate	−5	1	−0.317	0.177	0.149	−46,424.13	−46,421.11	−6.14	3.88
6	2,3-enediol	−4	1	−0.032	0.106	0.103	−46,437.75	−46,434.39	−7.65	3.32
7	2,3-enediolate	−5	1	0.048	−0.012	0.106	−46,424.27	−46,421.25	−5.97	4.14
8	BPGA	−4	1	0.002	0.068	0.075	−51,603.38	−51,599.04	−6.55	2.20
9	CABP	−4	1	−0.094	0.296	0.006	−51,603.53	−51,599.18	−8.09	2.45
10	3kCABP	−4	1	−0.122	0.254	0.339	−51,570.72	−51,567.08	−8.35	1.24
11	H_2_O	0	1	-	-	-	−2080.34	−2080.25	−11.36	3.50
12	H_3_O^+^	1	1	-	-	-	−2091.54	−2091.10	−15.95	2.38
13	CO_2_	0	1	0.474	-	-	−5132.52	−5132.76	−12.80	3.23

## Data Availability

https://www.researchgate.net/publication/354271173_Results_of_calculation.

## Appendix A

Figure A1, Figure A2, Figure A3, Figure A4, Figure A5, Figure A6, Figure A7, Figure A8, Figure A9, Figure A10 and Figure A11 in Appendix A show the optimized geometric structures of the compounds listed in Table 1. Bond lengths are indicated between atoms and Mulliken charges are indicated on the atoms. The total charge, Q; multiplicity, M; total energy, E_0_ and Gibbs free energy, G_0_ of each molecule are presented in the figure caption.
